# Large Language Models for Optimizing Patient Recruitment Decisions in Voice Data Generation Projects

**DOI:** 10.1002/lio2.70519

**Published:** 2026-08-24

**Authors:** James Anibal, Geetha Krishna Chaitanya Nama, Shrramana Ganesh, Yosef Nafii, Samantha Salvi Cruz, Veronica Daoud, Madeleine Zanin, Tram Le, Parsa Khorrami, Bradford J. Wood, David Clifton, Jamie Toghranegar, Yael Bensoussan, Yael E. Bensoussan, Olivier Elemento, Jean‐Christophe Bélisle‐Pipon, David Dorr, Satrajit Ghosh, Alistair Johnson, Phillip Payne, Maria E. Powell, Anaïs Rameau, Vardit Ravitsky, Alexandros Sigaras, Shaheen Awan, Ruth Bahr, Donald Bolser, Kathy J. Jenkins, Frank Rudzicz, Jennifer Siu, Stephanie Watts, Yassmeen Abdel‐Aty, Toufeeq Ahmed Syed, James Anibal, Steven Bedrick, Isaac Bevers, Micah Boyer, Rahul Brito, Selina A. Casalino, John Costello, Enrique Diaz‐Ocampo, Mahmoud Elmahdy, Kenneth Fletcher, Alexander Gelbard, Karim Hanna, Bill Hersh, Lochana Jayachandran, Kaley Jenney, Andrea Krussel, Chloe Loewith, Tempestt Neal, Claire Premi‐Bortolotto, Sarah Rohde, Samantha Salvi Cruz, Elizabeth Silberholz, Duncan Sutherland, Venkata Swarna Mukhi Talluri, Jamie Toghranegar, Kimberly Vinson, Claire Wilson, Madeleine Zanin, Theresa Zesiewicz, Robin Zhao

**Affiliations:** ^1^ Computational Health Informatics Lab, Institute of Biomedical Engineering University of Oxford Oxford UK; ^2^ Center for Interventional Oncology NIH Clinical Center, National Institutes of Health Bethesda Maryland USA; ^3^ Department of Computer Science and Engineering University of South Florida Tampa Florida USA; ^4^ USF Health Voice Center, Department of Otolaryngology‐Head & Neck Surgery University of South Florida Tampa Florida USA; ^5^ Vanderbilt University Medical Center Nashville Tennessee USA; ^6^ Mount Sinai Hospital Toronto Ontario Canada

**Keywords:** artificial intelligence, bias mitigation, dataset optimization, large language models

## Abstract

**Objectives:**

Past studies have shown that many clinical machine learning models have performance limitations due to imbalances in the training data. For voice data generation projects, the origin of the problem may lie in the recruiting methods used during data collection efforts. This study introduces a generative AI pipeline for “dataset decision support”, recommending recruitment decisions based on high‐dimensional insights.

**Methods:**

The publicly available GOSSIS‐1‐eICU dataset was filtered to create patient populations that were relevant to voice data generation projects. Lab results and vital signs from the electronic health record were also used to train a neural network for prediction of disease type. Prediction uncertainty estimates were included in the dataset as approximate indicators of health complexity. To select the best recruitment choice for addressing imbalances, an open‐source large language model (LLM) was then instructed to assess dataset statistics and the characteristics of possible participants. Simulations were run in which the system constructed datasets of 250 patients.

**Results:**

In over 90% of cases, the proposed system reduced categorical imbalances and widened continuous distributions when compared to randomly sampled counterfactual datasets (*q*‐value < 0.05). Variables included race, age, BMI, sex, disease type, oxygenation status, co‐morbidities, post‐operative status, Glasgow Coma Scale verbal response score, the Acute Physiology Score III, prediction uncertainty, vital signs, and lab results.

**Conclusion:**

LLMs may provide useful, explainable recommendations when presented with dataset distribution statistics and candidate profiles. In the future, this simulated scenario may be extended to align with conditions in emergency departments or other high‐volume settings.

**Level of Evidence:**

3.

## Graphical abstract

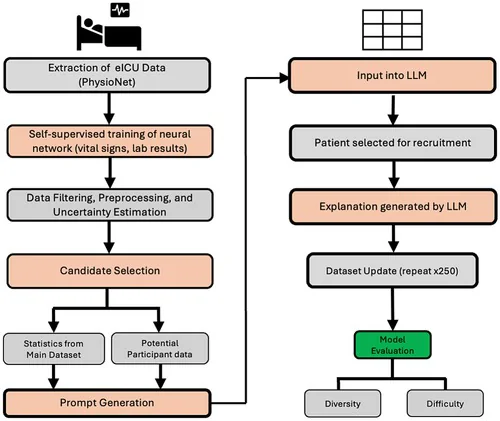

## 1 Introduction

Much like humans, artificial intelligence (AI) systems tend to perform worse on tasks that were not sufficiently “practiced” during the training process. Across many applications, studies have shown that these dataset imbalances (i.e., skewed distributions of sample attributes) might exacerbate disparities [1, 2]. This is particularly concerning for studies involving voice data. In many ways, voice AI models are distinct compared to other digital health tools, with a high sensitivity to accent, prosody, speech impairments, linguistic/cultural variability, and other factors that may be directly related to underrepresented attributes in the data [3, 4, 5, 6, 7, 8, 9, 10, 11, 12, 13, 14]. Many existing audio datasets contain significant (unintentional) class imbalances, and downstream bias has been mentioned as a major concern among stakeholders in voice AI projects [15, 16, 17, 18, 19].

### 1.1 Related Work

Currently, most bias mitigation techniques are in the form of new computational methods to improve AI model performance after data collection is complete [20, 21]. Early attempts were centered around simple data rebalancing and resampling, such as the synthetic minority oversampling technique (SMOTE) [22, 23, 24]. This algorithm, which has been widely used in practice, identifies the k‐nearest neighbors for a selected data point and then generates a synthetic sample via linear interpolation [22]. As the field has continued to progress, more sophisticated methods were designed that involved the direct adaptation of model learning objectives to prioritize underrepresented classes. Two examples of this include adversarial bias mitigation and reinforcement learning for algorithmic fairness [25, 26, 27, 28]. In the adversarial case, the objective is to maximize performance on a desired task (e.g., disease classification) while minimizing the ability of a discriminator model to identify sensitive variables like sex, race, or zip code. Both strategies were shown to reduce bias across different domain‐specific applications, including healthcare [25, 26, 27, 28]. Foundation models have also been developed for the task of rebalancing datasets through the creation of synthetic samples [29, 30]. One recent study used LLMs to identify attributes contributing to AI model bias and design targeted augmentations in the form of instructional prompts [31]. These textual outputs were then passed to a Stable Diffusion model that generated counterfactual training data, which led to systematic improvements in multiple fairness metrics [31]. While promising, these tools do not address the core problem of imbalanced data collection and are instead applied within the training process. At this later stage, robust bias mitigation is more challenging, creating an unsolved problem.

### 1.2 Voice Data Generation Projects

In voice data generation projects, harmful imbalances may result from improper curation. One major problem lies in flawed participant recruitment rationales during efforts to prospectively collect audio data. Such risks are particularly significant if the data generation project is aiming to collect a wide range of samples for generalist applications, such as (1) foundation model pre‐training, (2) the public release of a dataset (e.g., Bridge2AI‐Voice) for benchmarking purposes, or (3) tasks like severity and frailty assessments [13, 32, 33, 34, 35, 36, 37, 38]. Careful recruitment strategies may solve these issues but might also place unreasonable demands on researchers responsible for data collection, who would be required to do manual analysis of complex datasets. This would be particularly difficult in high‐volume or understaffed settings like emergency departments (EDs), where recruitment decisions must be made rapidly from large, diverse sets of possible participants. There have already been multiple early efforts to collect voice data from these environments, which may benefit greatly from fast, low‐cost, and non‐invasive diagnostic tools powered by audio biomarkers [39, 40]. However, to decrease the risk of subtle biases, solutions are needed to support data collection specialists who are operating in fast‐paced and often resource‐limited circumstances.

Large language models (LLMs) may present new opportunities to improve data collection processes within voice data generation projects. LLMs can rapidly parse textual and tabular inputs from multiple distinct sources, demonstrate advanced reasoning capabilities, provide outputs that are easy to understand, and respond to follow‐up questions. Foundation models can also adapt to variability in data structure, distribution types (i.e., categorical, continuous), noise, and missingness patterns. These capabilities may better address the broad range of factors that can contribute to training imbalances and poor generalization, potentially improving research outcomes in challenging recruitment settings like hospitals. In contrast, basic statistical methods, such as greedy inverse‐frequency sampling, are not as robust to data variations, are less easily aligned with continuous distributions, are neither interactive nor interpretable, and do not have advanced reasoning abilities. LLMs may be able to assess high‐dimensional data and recommend ideal recruitment choices.

However, further research is needed prior to deployment. Past work has illustrated concerns about biases and hallucinations within generative AI models that could impede the delivery of quality healthcare [41, 42, 43, 44]. Similar issues could have unexpected impacts on data collection processes as well. These prior results illustrate the need for studies that objectively characterize the potential of LLMs for “dataset decision support” within voice AI projects. While generative AI systems have been developed for automated tool selection, there is virtually no prior work on the use of LLMs for optimizing patient recruitment to achieve more balanced voice datasets [45].

The primary aim of this study was to determine if the application of LLMs led to a statistically significant reduction in class imbalance compared to baseline datasets. AI pipelines were designed to automate participant selection in a hypothetical voice data generation project similar to Bridge2AI‐Voice. Simulations were based on real‐world patient profiles that were extracted from the eICU subset of the GOSSIS dataset, including both demographic and clinical variables [46, 47]. This work introduces a novel LLM system for supporting clinical data collection specialists, a group often overlooked in the design and development of voice AI tools.

## 2 Materials and Methods

**FIGURE 1:**
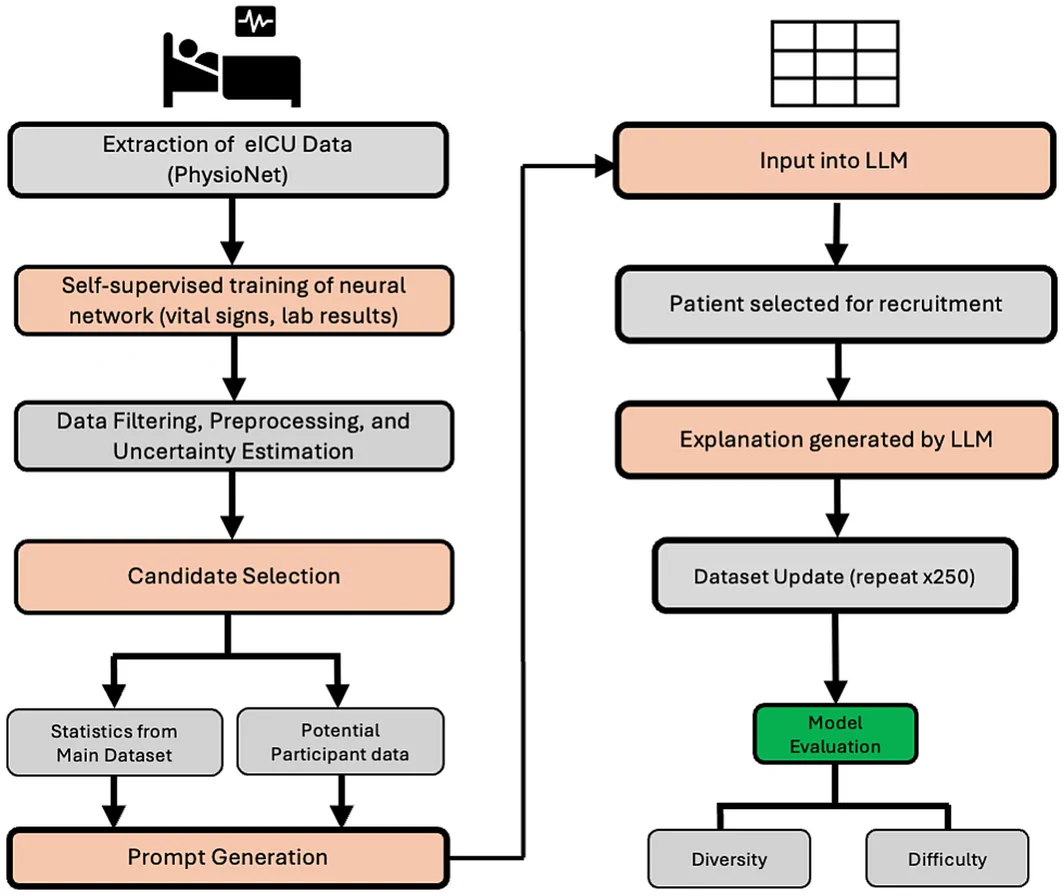
Workflow for evaluating the GPT‐OSS‐120B model in the task of selecting participants for a simulated voice data generation project. Steps include (1) extraction of EHR data from PhysioNet (GOSSIS‐1‐eICU), (2) Training of a neural network model for estimation of prediction uncertainties, (3) Filtering and preprocessing of EHR data to ensure an accurate simulation of a voice data generation project (e.g., removal of patients who would be unable to speak/produce coherent sound), (4) Curation of prompts for the LLM using class label statistics, distribution metrics, and potential candidates, (5) Generation of the LLM recommendation and explanation, and (6) Evaluation of the LLM recommendation.

The following section describes the datasets, preprocessing steps, and pipeline design methods for the simulation of LLM‐driven dataset decision support in a voice data generation project. Figure 1 provides a visual overview.

### 2.1 Datasets

This study utilized the eICU‐CRD subset of the Global Open‐Source Severity of Illness Score dataset (GOSSIS‐1‐eICU). The GOSSIS‐1‐eICU dataset, which is hosted on the PhysioNet platform, consists of 131,051 deidentified electronic health records collected from patients during the first 24 h of ICU admission [46, 47]. GOSSIS‐1‐eICU data are categorized based on the following disease group labels: Cardiovascular, Neurological, Trauma, Sepsis, Genitourinary, Gastrointestinal, Respiratory, Metabolic, Hematological, Musculoskeletal/Skin, Gynecological, and Other Medical Disorders.

### 2.2 Neural Network for Uncertainty Representation

To ensure the recruitment simulation would include clinical variables beyond known sources of bias and paralinguistic variability (Tables 1 and 2), a neural network was trained to encode 52 EHR features (Table 3) using disease group prediction as the representation learning objective [74, 75]. Disease group was chosen for this study due to the emphasis on dataset optimization: for the goal of ensuring diverse patient recruitment, ideal representations should encode nuanced interactions between different body systems. Model uncertainties could then be used as low‐dimensional representations of complex health states, reducing the risk of downstream LLM performance degradation caused by overly lengthy inputs [76].

**TABLE 1:** Description of selected categorical variables with a potential or known risk of introducing imbalances or confounding factors into the training datasets of voice AI models.

Variables	Description	Impact on voice datasets
Age	Chronological age of the patient at the time of data collection (in years).	Older patients may have voice changes that occur naturally during the aging process [48, 49, 50].
Race/Ethnicity	Self‐identified racial or ethnic group of the patient.	Different ethnic groups may have varying accents or experience diseases differently, resulting in both linguistic and paralinguistic variation [48, 49, 50].
Sex	Sex assigned at birth (recorded as male or female).	Multiple studies have found voice differences between sexes, including from sex‐specific hormonal changes (e.g., monthly menstrual cycles) [48, 49, 50].
Diagnosis (by System)	Primary diagnosis grouped by affected organ system or clinical syndrome (e.g., cardiovascular, respiratory, neurological).	Voice and speech biomarkers may vary widely across disease categories (e.g., between neurological and respiratory illnesses). Moreover, a diverse set of diagnoses may help ensure sufficient breadth and depth within the control cohort [48, 49, 50].
Oxygenation Status	Clinical measure of the patient's oxygenation level and support, such as room air or supplemental oxygen.	Different oxygenation statuses may cause voice changes, such as increased hoarseness, that arise as a direct result of the intervention [51].
Glasgow Coma Scale Verbal Score (GCS‐verbal)	Score reflecting level of consciousness based on verbal responses.	Lower GCS Verbal Scores are associated with decreased coherence of speech [9, 10, 11, 12, 52].
Body Mass Index (BMI)	Anthropometric index calculated as weight in kilograms divided by height in meters squared (kg/m^2^).	Studies have found evidence of voice changes related to obesity, such as reduced lung capacity or fat accumulation in the neck and pharyngeal tissues [53, 54, 55].
Acute Respiratory Failure Status	Indicator of whether the patient has a documented diagnosis of acute respiratory failure.	Acute respiratory failure may be associated with speech and voice changes due to impaired oxygenation, increased work of breathing, dyspnea, and reduced breath support, which can cause altered vocal quality, decreased phonation time, and vocal fatigue [56, 57, 58].
Diabetes Mellitus	Indicator of whether the patient has a documented diagnosis of diabetes.	Studies have shown that diabetes may cause voice changes due to factors like diabetic neuropathy, microvascular injury, and myopathic changes that may affect laryngeal neuromuscular control, vocal fold vibration, and respiratory support for phonation [59].
Immunosuppression	Indicator of whether the patient has received an intervention resulting in suppression of the immune system, including chemotherapy and radiation therapy.	Immunosuppression may be associated with voice changes, particularly through increased susceptibility to laryngeal infections, such as fungal laryngitis, and effects related to medications [60, 61, 62].
Post‐Operative Status	Indicator of whether the patient is in the period following a surgical procedure.	Post‐operative patients may experience voice changes that are due to side effects of intubation or nerve damage [63].

**TABLE 2:** Description of selected continuous variables with a potential or known risk of introducing imbalances or confounding factors into the training datasets of voice AI models.

Variables	Description	Impact on voice datasets	Missingness
Maximum Heart Rate	Maximum recorded heart rate during the initial 24‐h period, measured in beats per minute (BPM)	Elevated heart rate may reflect acute cardiovascular stress, tachyarrhythmia, or reduced cardiopulmonary reserve, which can affect phonation through increased respiratory effort, dyspnea, and vocal fatigue [64, 65].	0.22%
Minimum Mean Blood Pressure	Minimum recorded mean blood pressure during the initial 24‐h period, measured in millimeters of mercury (mmHg).	Abnormal blood pressure may relate to voice changes through cardiovascular and respiratory mechanisms, including altered breath support, cardiopulmonary strain, and changes in speech acoustics [66].	0.60%
Maximum Respiratory Rate	Maximum recorded respiratory rate during the initial 24‐h period, measured in breaths per minute.	Respiratory distress may impact acoustic and prosodic features of speech and voice, including changes in voice quality, speaking pattern, loudness, speech rate, and pause duration [56, 57, 58].	4.01%
Minimum Peripheral Capillary Oxygen Saturation (SpO_2_)	Minimum recorded peripheral oxygen saturation during the initial 24‐h period, measured as a percentage.	Reduced oxygenation and hypoxemia may be associated with changes in voice quality. Greater time spent below 90% oxygen saturation has been associated with worsening acoustic voice parameters [56, 57, 58].	0.97%
Maximum Blood Urea Nitrogen (BUN)	Maximum recorded blood urea nitrogen during the initial 24‐h period, measured in milligrams per deciliter (mg/dL).	Renal dysfunction may be associated with measurable changes in acoustic and aerodynamic voice characteristics caused by fatigue, altered respiratory support, fluid imbalance, and neuromuscular effects [67, 68].	12.33%
Maximum Bilirubin	Maximum recorded bilirubin during the initial 24‐h period, measured in milligrams per deciliter (mg/dL).	Hepatic failure, particularly when complicated by hepatic encephalopathy, may be associated with voice and speech changes such as dysarthria, reduced speech clarity, and altered vocal quality [69, 70].	61.61%
Maximum Lactate	Maximum recorded lactate during the initial 24‐h period, measured in millimoles per liter (mmol/L).	Tissue hypoperfusion, anaerobic metabolism, and increased severity of acute illness, which are indicated by elevated lactate, may be associated with voice changes through respiratory distress, reduced cardiopulmonary reserve, fatigue, and altered breathing patterns [71, 72].	80.35%
Acute Physiology Score III (APSIII)	Acute Physiology Score is a severity score derived from the most abnormal physiologic measurements during the initial 24‐h period.	Datasets with disease severity imbalances may lead to voice AI models that cannot perform nuanced triage tasks. For example, all mildly ill patients may be equally labeled as “healthy” [73].	10.03%

**TABLE 3:** GOSSIS‐1‐eICU variables used as the basis for training the neural network for uncertainty estimation, as described in Section 2.2.

Variable name	Description
d1_diasbp_avg	Midpoint between the minimum and maximum diastolic blood pressure recorded during the first 24 h after ICU admission.
d1_heartrate_avg	Midpoint between the minimum and maximum heart rate recorded during the first 24 h after ICU admission.
d1_mbp_avg	Midpoint between the minimum and maximum mean arterial blood pressure recorded during the first 24 h after ICU admission.
d1_resprate_avg	Midpoint between the minimum and maximum respiratory rate recorded during the first 24 h after ICU admission.
d1_spo2_avg	Midpoint between the minimum and maximum peripheral oxygen saturation (SpO_2_) recorded during the first 24 h after ICU admission.
d1_sysbp_avg	Midpoint between the minimum and maximum systolic blood pressure recorded during the first 24 h after ICU admission.
d1_temp_avg	Midpoint between the minimum and maximum body temperature recorded during the first 24 h after ICU admission.
d1_albumin_avg	Midpoint between the minimum and maximum serum albumin concentration recorded during the first 24 h after ICU admission.
d1_bilirubin_avg	Midpoint between the minimum and maximum serum bilirubin concentration recorded during the first 24 h after ICU admission.
d1_bun_avg	Midpoint between the minimum and maximum blood urea nitrogen (BUN) concentration recorded during the first 24 h after ICU admission.
d1_creatinine_avg	Midpoint between the minimum and maximum serum creatinine concentration recorded during the first 24 h after ICU admission.
d1_glucose_avg	Midpoint between the minimum and maximum blood glucose concentration recorded during the first 24 h after ICU admission.
d1_hco3_avg	Midpoint between the minimum and maximum serum bicarbonate (HCO_3_^−^) concentration recorded during the first 24 h after ICU admission.
d1_hemaglobin_avg	Midpoint between the minimum and maximum hemoglobin concentration recorded during the first 24 h after ICU admission.
d1_hematocrit_avg	Midpoint between the minimum and maximum hematocrit value recorded during the first 24 h after ICU admission.
d1_inr_avg	Midpoint between the minimum and maximum international normalized ratio (INR) of prothrombin time recorded during the first 24 h after ICU admission.
d1_lactate_avg	Midpoint between the minimum and maximum blood lactate concentration recorded during the first 24 h after ICU admission.
d1_platelets_avg	Midpoint between the minimum and maximum platelet count recorded during the first 24 h after ICU admission.
d1_potassium_avg	Midpoint between the minimum and maximum serum potassium concentration recorded during the first 24 h after ICU admission.
d1_sodium_avg	Midpoint between the minimum and maximum serum sodium concentration recorded during the first 24 h after ICU admission.
d1_calcium_avg	Midpoint between the minimum and maximum serum calcium concentration recorded during the first 24 h after ICU admission.
d1_wbc_avg	Midpoint between the minimum and maximum white blood cell (WBC) count recorded during the first 24 h after ICU admission.
d1_arterial_pco2_avg	Midpoint between the minimum and maximum arterial partial pressure of carbon dioxide (PaCO_2_) recorded during the first 24 h after ICU admission.
d1_arterial_ph_avg	Midpoint between the minimum and maximum arterial blood pH recorded during the first 24 h after ICU admission.
d1_arterial_po2_avg	Midpoint between the minimum and maximum arterial partial pressure of oxygen (PaO_2_) recorded during the first 24 h after ICU admission.
d1_pao2fio2ratio_avg	Midpoint between the minimum and maximum ratio of arterial partial pressure of oxygen to inspired oxygen fraction (PaO_2_/FiO_2_) recorded during the first 24 h after ICU admission.
d1_diasbp_diff	Range (maximum minus minimum) of diastolic blood pressure recorded during the first 24 h after ICU admission, representing variability in diastolic blood pressure.
d1_heartrate_diff	Range (maximum minus minimum) of heart rate recorded during the first 24 h after ICU admission, representing variability in heart rate.
d1_mbp_diff	Range (maximum minus minimum) of mean arterial blood pressure recorded during the first 24 h after ICU admission, representing variability in mean arterial pressure.
d1_resprate_diff	Range (maximum minus minimum) of respiratory rate recorded during the first 24 h after ICU admission, representing variability in respiratory rate.
d1_spo2_diff	Range (maximum minus minimum) of peripheral oxygen saturation (SpO_2_) recorded during the first 24 h after ICU admission, representing variability in oxygen saturation.
d1_sysbp_diff	Range (maximum minus minimum) of systolic blood pressure recorded during the first 24 h after ICU admission, representing variability in systolic blood pressure.
d1_temp_diff	Range (maximum minus minimum) of body temperature recorded during the first 24 h after ICU admission, representing variability in temperature.
d1_albumin_diff	Range (maximum minus minimum) of serum albumin concentration recorded during the first 24 h after ICU admission, representing variability in albumin levels.
d1_bilirubin_diff	Range (maximum minus minimum) of serum bilirubin concentration recorded during the first 24 h after ICU admission, representing variability in bilirubin levels.
d1_bun_diff	Range (maximum minus minimum) of blood urea nitrogen (BUN) concentration recorded during the first 24 h after ICU admission, representing variability in BUN levels.
d1_creatinine_diff	Range (maximum minus minimum) of serum creatinine concentration recorded during the first 24 h after ICU admission, representing variability in creatinine levels.
d1_glucose_diff	Range (maximum minus minimum) of blood glucose concentration recorded during the first 24 h after ICU admission, representing variability in glucose levels.
d1_hco3_diff	Range (maximum minus minimum) of serum bicarbonate (HCO_3_^−^) concentration recorded during the first 24 h after ICU admission, representing variability in bicarbonate levels.
d1_hemaglobin_diff	Range (maximum minus minimum) of hemoglobin concentration recorded during the first 24 h after ICU admission, representing variability in hemoglobin levels.
d1_hematocrit_diff	Range (maximum minus minimum) of hematocrit value recorded during the first 24 h after ICU admission, representing variability in hematocrit.
d1_inr_diff	Range (maximum minus minimum) of international normalized ratio (INR) of prothrombin time recorded during the first 24 h after ICU admission, representing variability in INR.
d1_lactate_diff	Range (maximum minus minimum) of blood lactate concentration recorded during the first 24 h after ICU admission, representing variability in lactate levels.
d1_platelets_diff	Range (maximum minus minimum) of platelet count recorded during the first 24 h after ICU admission, representing variability in platelet count.
d1_potassium_diff	Range (maximum minus minimum) of serum potassium concentration recorded during the first 24 h after ICU admission, representing variability in potassium levels.
d1_sodium_diff	Range (maximum minus minimum) of serum sodium concentration recorded during the first 24 h after ICU admission, representing variability in sodium levels.
d1_calcium_diff	Range (maximum minus minimum) of serum calcium concentration recorded during the first 24 h after ICU admission, representing variability in calcium levels.
d1_wbc_diff	Range (maximum minus minimum) of white blood cell (WBC) count recorded during the first 24 h after ICU admission, representing variability in WBC count.
d1_arterial_pco2_diff	Range (maximum minus minimum) of arterial partial pressure of carbon dioxide (PaCO_2_) recorded during the first 24 h after ICU admission, representing variability in PaCO_2_.
d1_arterial_ph_diff	Range (maximum minus minimum) of arterial blood pH recorded during the first 24 h after ICU admission, representing variability in arterial pH.
d1_arterial_po2_diff	Range (maximum minus minimum) of arterial partial pressure of oxygen (PaO_2_) recorded during the first 24 h after ICU admission, representing variability in PaO_2_.
d1_pao2fio2ratio_diff	Range (maximum minus minimum) of the ratio of arterial partial pressure of oxygen to inspired oxygen fraction (PaO_2_/FiO_2_) recorded during the first 24 h after ICU admission, representing variability in the oxygenation ratio.

Given the tabular data structure, a simple multi‐layer network architecture was selected to maximize computational efficiency and reduce the risk of overfitting. The model was trained, validated, and tested on a set of 30,000 patients sampled randomly from the “model‐ready” version of GOSSIS‐1‐eICU, which addressed data missingness through imputation techniques. These data points were removed from downstream recruitment simulations. The cross‐entropy loss function was used to evaluate model error on minibatches of scaled data (0–1 range). Weight updates were calculated via the Adam optimization function. The neural network was trained for a maximum of 50 epochs, with early stopping based on validation loss. The dropout rate of the model was set to 0.5. After training, the model yielded a cross‐validated “one‐vs‐rest” AUC score of 0.88, indicating that the outputs were reasonably reflective of real‐world disease states. Consequently, Monte Carlo uncertainty estimates were considered to be approximate indicators of patients with “fuzzy” health conditions. Inference was performed across 100 iterations, with random dropout activated throughout the process [77]. The normalized per‐class uncertainty was then obtained by applying the softmax transformation and calculating the standard deviation of the repeated predictions [77]. Many current voice datasets are skewed in favor of patients with clearly characterized health statuses that easily facilitate the training of supervised AI models. The uncertainties of machine learning algorithms, if factored into participant recruitment strategies, may help ensure that training datasets have a diverse range of clinical profiles. This is a known limitation of past voice AI studies [78].

### 2.3 Data Preprocessing

After removing the patient records used for training the uncertainty estimation network described in Section 2.2, additional preprocessing steps were necessary to ensure an accurate simulation of a voice data generation project. First, 12 categorical and 8 continuous variables were chosen for inclusion in the study (Tables 1 and 2). These represented a source of bias in past work or were previously associated with paralinguistic variability that could impact the performance of audio AI models if underrepresented during the training phase [48, 49, 50, 51, 52, 53, 54, 55, 56, 57, 58, 59, 60, 61, 62, 63, 64, 65, 66, 67, 68, 69, 70, 71, 72, 73, 79]. For continuous variables with multiple available measurements in the initial 24‐h period, such as laboratory test results and recorded vital signs, either the minimum or maximum value was used depending on the approximate orientation of clinical severity.

To ensure that each candidate could be compared to the simulated exclusion criteria (described below), samples were filtered if any categorical variable was missing from the data profile. The Glasgow Coma Scale verbal response score (GCS‐verbal) was further used to exclude any patients who would be incapable of producing coherent sound. Only adult, non‐ventilated patients with GCS‐verbal scores of 4 or 5 (confused and oriented, respectively) were considered in this study. Patients with a GCS‐verbal score lower than 4 would likely be unable to complete a protocol due to extreme disorientation or an inability to speak. Age and BMI were binned prior to inclusion in the experiments, reducing complexity (see Table 4). Binning strategies were selected based on recommendations from leading public health agencies and precedent from past research [80, 81, 82]. Within each sample, missing continuous variables and obvious artifacts (e.g., a respiratory rate of 100) were labeled as “No Value”, preserving real‐world data sparsity patterns that may be observed in future deployment environments. Missingness rates for each continuous variable can be found in Table 2.

**TABLE 4:** Binning strategies used for the data preprocessing pipeline presented in this study.

Variable name	Bin	Explanation
Age	18–24	Late adolescence/Emerging adulthood
Age	25–39	Early adulthood
Age	40–64	Midlife
Age	65+	Older adulthood
BMI	0–18.49	Underweight
BMI	18.5–24.9	Healthy weight
BMI	25–29.9	Overweight
BMI	30–34.9	Class 1 obesity
BMI	35–39.9	Class 2 obesity
BMI	40+	Class 3 (Severe) obesity

Upon completion of the steps described above, the preprocessed subset of the GOSSIS‐1‐eICU dataset contained electronic health records from 55,589 patients. Table 5 shows demographic statistics for the dataset. Alongside the bias variables in Tables 1 and 2, the neural network prediction uncertainties for each sample (*n* = 55,589) were included in the dataset. To generate these values, the neural network model (Section 2.2) performed inference on the data via the Monte Carlo method, and the per‐class uncertainties were then binarized into high‐ or low‐confidence predictions. The threshold was set to the 90th percentile of uncertainty values in the validation data. The total count of uncertain predictions (exceeding the threshold) for each patient could then be calculated and binned into two groups: stable (< 2/12 uncertain predictions) and fuzzy (≥ 2/12 uncertain predictions).

**TABLE 5:** Demographic and clinical statistics for the subset of the GOSSIS‐1‐eICU database that was curated for this study.

Class	Prevalence
Age group: 18–24	0.031
Age group: 25–39	0.092
Age group: 40–64	0.395
Age group: 65+	0.482
Race: Caucasian	0.775
Race: Black/African American	0.110
Race: Asian	0.017
Race: Hispanic	0.041
Race: Native American	0.007
Race: Other/Unknown	0.051
Sex: Female	0.458
Sex: Male	0.542
GCS verbal score: 4	0.152
GCS verbal score: 5	0.848

### 2.4 LLM Methods

This study involved the use of a large language model (LLM) to enhance the balance of simulated voice datasets collected prospectively through participant recruitment (Figure 1). Experiments were performed using the GPT‐OSS open‐source large language model (120 billion parameters) through the DeepInfra standard inference API [83, 84]. This choice was made to ensure the protection of patient data and privacy in accordance with PhysioNet policies for credentialed access. DeepInfra does not retain any input data on disk; requests are held only in memory during the inference process. When tested on benchmark datasets, GPT‐OSS achieved comparable levels of performance to proprietary models like GPT‐4o, Claude Sonnet, or Gemini, providing an ideal balance between security and capability [84, 85].

#### 2.4.1 Application of the LLM

First, a prompt was designed to instruct the LLM on how to recommend candidates (see Table 6). The model was told to consider the complete data profiles, including binned uncertainty values from the supervised neural network. For the purposes of this study, GPT‐OSS was instructed to optimize for absolute balance within the dataset (i.e., equal prevalence of class labels for each categorical attribute and maximally wide distributions for continuous variables), which is important for the development of public repositories and/or the pre‐training of foundation models. In future applications, the definition of balance might differ depending on the goals of the study (e.g., research on vocal cord paralysis may be focused on maximizing diversity within post‐operative patients). There may be specific circumstances where imbalances are intentional, such as a study to understand voice changes in menopausal women, but this work focuses on the general case (i.e., building a dataset for public release or use as a pre‐training corpus) [86].

**TABLE 6:** LLM prompt (exactly as used in the experiments, broken down by key sections).

Section	Text from prompt	Purpose
Task	Automate participant selection for a voice data generation project aimed at enhancing the performance of voice AI models. Select the single best candidate patient from the list below whose comprehensive clinical profile is most informative for ensuring a balanced voice AI training dataset.	Defines the general parameters of the model task
Context	Voice and speech production can be affected by many clinical states. These should be included in a balanced voice AI training dataset (examples below). Impaired airway protection: Post‐operative state. Respiratory pathology or compromised gas exchange: Acute respiratory failure, supplemental oxygen requirement, elevated respiratory rate, low SpO_2_, respiratory diagnosis category. Neurologic illness or altered consciousness: Neurologic or trauma diagnosis category, low GCS verbal score. Severe systemic illness affecting vocal fold tissue or neuromuscular function: High acute physiology score, tachycardia, abnormally low MBP indicating hypotension, sepsis diagnosis category, elevated lactate, elevated BUN, elevated bilirubin. Chronic comorbidities with known voice or speech impact: Diabetes mellitus, immunosuppression.	Provides examples of how different variables impact the variability of voice datasets
Criteria	Reason over the full candidate profile jointly. Jointly interpret continuous and categorical variables. Categorical features help provide clinical interpretation of continuous values. For example, a lactate of 4.0 in a patient with Sepsis has a different clinical meaning than the same lactate value recorded post‐operatively. Ideal candidates have categorical and continuous variables that help address gaps in the existing voice dataset. Use the categorical class distribution and the continuous distributional summary below to identify which clinical profiles are currently underrepresented in the existing dataset. Do not consider any external or pre‐existing knowledge about bias in AI datasets. Important: Ignore the distribution of attributes within the list of potential candidates. Evaluate each profile as if the candidate was sampled from a balanced set. Decisions should be based only on how each candidate would impact the existing dataset.	Outlines the decision‐making criteria of the LLM
Inputs	Data Format: Each vital sign and laboratory value is reported as the most clinically informative extreme recorded in the past 24 h. This is indicated by the _max or _min suffix on the feature name (e.g., d1_lactate_max is the peak lactate, d1_spo2_min is the lowest oxygen saturation). If a lab or vital was not recorded for a patient, the value is the string “No Value”. “No Value” is true missing data; do not impute. *Existing Dataset Balance (Categorical)* Per‐variable max‐min class proportion gap (pp) in the existing dataset, grouped by clinical domain. Higher gap = more imbalanced. {Variable Proportion Gaps} *Existing Dataset Class Distributions (Categorical)* Counts of each categorical variable class in the existing dataset. Use these to determine which clinical categories are underrepresented and would most affect a voice AI training dataset {Categorical Variable Counts} *Existing Dataset Distributional Summary (Continuous)* For each continuous feature, the existing dataset's distributional statistics: *n* (current sample size), mean, std., min, max, median, q10, q25, q75, q90. q10 and q90 mark the lower‐ and upper‐decile boundaries. Continuous Distribution Optimization The selection should produce a dataset that captures the full range of clinically distinct presentations for each continuous feature, ranging from fully normal to extremely abnormal. The dataset should represent the pathophysiology that occurs in real patients across the full spectrum of severity. Distributions should not be skewed in either direction, but should be clinically complete. Use these statistics and your clinical knowledge to identify gaps in the existing dataset that reduce clinical completeness. These may include undersampled ranges and skewed distributions, which could impact the breadth of voice data available for the training of AI models. {Continuous Distribution Statistics} Candidate Patients (Full Sample Pool) Key requirement: Do not consider balance within this pool when analyzing the data profiles. Evaluate each profile as if the candidate was sampled from a balanced set. Decisions should be based only on the existing dataset. {Dataset of *n* possible candidates for recruitment}	Describes the data that will be shown to the LLM.
Outputs	Return a single Python dictionary literal with exactly these keys “Index”: integer—The exact Index of the chosen candidate from the list above. “Profile_Rationale”: string—Clearly state the rationale for this decision. Additionally, clearly state how the selected candidate will improve paralinguistic diversity in the dataset. Examples of this could include Variations in speech coherence (e.g., GCS verbal score) Age‐related vocal characteristics Clinical factors affecting voice or speech (e.g., respiratory or neurological conditions) Return ONLY the Python dictionary literal No additional text, headings, commentary, markdown formatting, or bullet points. Do NOT wrap the dictionary in backticks or include any labels.	Provides specific instructions for the LLM response

The LLM was then used to simulate the process of voice data collection. The temperature parameter of the model was set to zero, facilitating reproducibility and ensuring that all outputs were aligned with the internal knowledge encodings of the model. Due to the complexity of the task, the reasoning level of the model was set to medium (default value is none). Fifty (50) patient profiles were sampled without replacement from the base dataset (GOSSIS‐1‐eICU), representing potential participants who may be available at a given point in time. The sample size was chosen to approximately simulate a high‐volume setting, such as a large hospital, where difficult recruitment decisions must be made quickly. To reduce dimensionality and compute requirements, the current state of the dataset was summarized by calculating the frequency of class labels and the distributional metrics of continuous variables. For example, if the EHR contained the variable “sex at birth”, the LLM input would be the numbers of male and female subjects in the current simulated dataset. Distributions of vital sign measurements and laboratory test results were represented through a set of statistical metrics, including current sample size (*n*), mean, median, standard deviation, minimum, maximum, interquartile range, 10th percentile, 25th percentile, 75th percentile, and 90th percentile. This cost‐effective method tested the capacity of LLMs to infer possible representation gaps even without access to the full dataset. The model outputs included a final recruitment recommendation and an explanation of how the candidate contributes to the clinical voice diversity of the dataset (Table 6). This may support researchers in clearly understanding the “audiomic” implications of participant selection, allowing for follow‐up questions, transparent reporting, and precise adjustments.

After each recruitment decision, the existence of the patient was verified by cross‐referencing the output with the original dataset, accounting for potential LLM hallucinations or artifacts when returning the index of recommended candidates. Following this step, the “new” dataset was updated with the selected candidate, and distribution statistics were recalculated prior to the next iteration. This process was repeated 250 times (the model evaluated 250 sets of 50 candidates). In total, 25 separate datasets were generated (each with *n =* 250), ensuring that statistical testing could be used to evaluate model performance. All results shown represent the mean outcome across the 25 experiments.

## 3 Results

Results are presented as a comparison of the LLM‐optimized datasets (25 datasets of 250 patients) and 500 randomly sampled counterfactuals (*n* = 250). For each discrete attribute level (e.g., male, female), the balance metric was defined as the observed proportion of patients with that value, computed separately within the 25 LLM‐optimized datasets and the 500 counterfactual cohorts. The balance of continuous variables was measured through the interdecile range (IDR). The differences between the LLM and counterfactual datasets were assessed using a two‐sided Mann–Whitney *U*‐test applied to both the categorical attribute levels with non‐zero prevalence and the IDR values (49 in total). The resulting *p*‐values were adjusted using the Benjamini–Hochberg false discovery rate (FDR) procedure. This approach controls the expected proportion of false positives among results deemed significant following many tests. All *p*‐values were ranked from smallest to largest and converted to FDR‐adjusted values (*q*‐values); significance was defined as *q* < 0.05, corresponding to an expected false discovery proportion of approximately 5% among the significant differences.

Despite missing data, the LLM pipeline was able to consistently narrow the range of class prevalence values compared to baseline while also expanding the distribution of continuous variables. On average, the model required less than 1 min to generate the recommendations, all of which included valid patient indices. After FDR correction, 46 of 49 comparisons were found to be statistically significant (*q* < 0.05), indicating systematic differences between the LLM‐optimized and random cohorts. As shown in Table 7, significant demographic shifts were observed in the age and ethnicity groups, including increases across African American, Asian, Hispanic, Native American, and Other/Unknown classes. Younger age categories (18–24 and 25–39) were also more prevalent in the LLM‐optimized cohorts, correcting for the overrepresentation of older patients in hospital settings. Clinical composition also improved when model recommendations were used to curate the simulated datasets. The Acute Respiratory Failure and GCS Verbal Score variables showed a clear redistribution towards severe cases, potentially indicating the inclusion of more participants with labored or confused speech (thereby helping ensure that downstream models are robust to these potential confounders). For the chronic co‐morbidity and disease group categories, all variables were more balanced in the LLM‐optimized datasets. There was also a higher prevalence of patients with an uncertain neural network prediction (Section 2.2), offering a potential solution to biases against profiles that cannot be easily labeled by conventional medical definitions.

**TABLE 7:** Comparison of categorical class balances between LLM‐optimized datasets and 500 counterfactual random sample datasets (from the GOSSIS‐1‐eICU subset considered in the study).

Attribute	Value	LLM mean ± SD (%)	Random mean ± SD (%)	Effect size (pp)	*q*
Age	18–24	6.8 ± 1.2	3.1 ± 1.1	+3.7	< 0.0001
Age	25–39	11.8 ± 1.8	9.2 ± 1.8	+2.7	< 0.0001
Age	40–64	36.7 ± 2.7	39.4 ± 3.0	−2.7	< 0.0001
Age	65+	44.6 ± 1.7	48.3 ± 3.0	−3.7	< 0.0001
BMI	0–18.49	8.6 ± 1.6	4.8 ± 1.3	+3.7	< 0.0001
BMI	18.5–24.9	29.7 ± 3.1	28.0 ± 2.9	+1.8	0.0062
BMI	25–29.9	27.8 ± 3.4	30.7 ± 2.9	−2.9	0.0001
BMI	30–34.9	16.1 ± 2.2	19.0 ± 2.5	−2.9	< 0.0001
BMI	35–39.9	8.7 ± 1.4	9.1 ± 1.9	−0.4	0.3085
BMI	40+	9.0 ± 1.3	8.3 ± 1.8	+0.7	0.0324
Ethnicity	African American	19.2 ± 2.1	11.0 ± 2.0	+8.2	< 0.0001
Ethnicity	Asian	8.6 ± 1.1	1.7 ± 0.8	+6.9	< 0.0001
Ethnicity	Caucasian	45.2 ± 2.1	77.4 ± 2.7	−32.2	< 0.0001
Ethnicity	Hispanic	9.6 ± 0.9	4.1 ± 1.2	+5.5	< 0.0001
Ethnicity	Native American	5.9 ± 1.1	0.7 ± 0.5	+5.2	< 0.0001
Ethnicity	Other/Unknown	11.4 ± 1.5	5.1 ± 1.4	+6.4	< 0.0001
Gender	F	48.1 ± 2.4	45.7 ± 3.2	+2.4	0.0003
Gender	M	51.9 ± 2.4	54.3 ± 3.2	−2.4	0.0003
GCS verbal	4	37.6 ± 1.9	15.2 ± 2.2	+22.3	< 0.0001
GCS verbal	5	62.4 ± 1.9	84.8 ± 2.2	−22.3	< 0.0001
Body system	Cardiovascular	20.3 ± 2.5	32.4 ± 2.9	−12.1	< 0.0001
Body system	Gastrointestinal	11.1 ± 1.9	11.6 ± 2.0	−0.5	0.2510
Body system	Genitourinary	5.5 ± 1.0	2.9 ± 1.1	+2.6	< 0.0001
Body system	Metabolic	9.0 ± 1.6	10.0 ± 1.8	−1.0	0.0040
Body system	Neurological	10.4 ± 1.3	14.3 ± 2.1	−3.9	< 0.0001
Body system	Other	5.2 ± 1.3	2.8 ± 1.1	+2.4	< 0.0001
Body system	Respiratory	12.5 ± 1.6	9.3 ± 1.8	+3.2	< 0.0001
Body system	Sepsis	21.5 ± 2.3	11.8 ± 2.1	+9.7	< 0.0001
Body system	Trauma	4.5 ± 0.7	4.9 ± 1.3	−0.4	0.1283
Respiratory failure (ARF)	0	74.7 ± 1.2	97.1 ± 1.0	−22.4	< 0.0001
Respiratory failure (ARF)	1	25.3 ± 1.2	2.9 ± 1.0	+22.4	< 0.0001
FiO_2_	0	74.5 ± 1.3	94.6 ± 1.4	−20.2	< 0.0001
FiO_2_	1	25.5 ± 1.3	5.4 ± 1.4	+20.2	< 0.0001
Diabetes	0	66.8 ± 1.3	78.0 ± 2.5	−11.2	< 0.0001
Diabetes	1	33.2 ± 1.3	22.0 ± 2.5	+11.2	< 0.0001
Postoperative	0	74.4 ± 1.1	83.3 ± 2.4	−8.9	< 0.0001
Postoperative	1	25.6 ± 1.1	16.7 ± 2.4	+8.9	< 0.0001
Immunosuppression	0	72.0 ± 1.2	97.0 ± 1.1	−25.1	< 0.0001
Immunosuppression	1	28.0 ± 1.2	3.0 ± 1.1	+25.1	< 0.0001
Physiological uncertainty	0	59.2 ± 2.3	73.1 ± 2.8	−13.9	< 0.0001
Physiological uncertainty	1	40.8 ± 2.3	26.9 ± 2.8	+13.9	< 0.0001

*Note:* The values are shown as proportions of the overall dataset in percentage form.

Among the continuous variables, similar results were observed between the LLM‐optimized and random counterfactual datasets. In particular, the Apache III score (APSIII) distribution was broadened by over 30%. This increased the representation of patients with higher disease severity, which is necessary to develop voice AI models for non‐invasive patient monitoring tasks, particularly in emerging applications such as prediction of cardiopulmonary decompensation [13, 33, 34, 35, 36, 37, 87]. The interdecile ranges for the other vital signs and laboratory values were also significantly expanded (effect sizes between 12% and 110%), showing that the LLM may be able to recommend patients ranging from normal physiological function to acute clinical states that may be associated with voice changes. These include congestive heart failure, hepatic encephalopathy, uremic delirium, fluid imbalances, dehydration, hypoxemia, or hypotension. All results for the continuous variables are reported in Table 8.

**TABLE 8:** Comparison of continuous variable distributions between LLM‐optimized datasets and 500 counterfactual random sample datasets (from the GOSSIS‐1‐eICU subset considered in the study).

Attribute	LLM IDR ± SD	Random IDR ± SD	Effect size (%)	*q*
Max HR (bpm)	62.4 ± 3.55	53.3 ± 2.97	+17%	< 0.0001
Min MBP (mmHg)	41.9 ± 2.16	37.5 ± 2.41	+12%	< 0.0001
Max Resp rate (br/min)	20.1 ± 1.05	16.9 ± 1.43	+19%	< 0.0001
Min SpO_2_ (%)	19.4 ± 1.91	11.2 ± 1.13	+72%	< 0.0001
Max BUN (mg/dL)	64.3 ± 5.75	40.6 ± 4.89	+58%	< 0.0001
Max Bilirubin (mg/dL)	3.54 ± 0.58	1.69 ± 0.46	+110%	< 0.0001
Max Lactate (mmol/L)	6.41 ± 1.03	3.22 ± 0.85	+99%	< 0.0001
APSIII (points)	46.9 ± 3.15	35.3 ± 2.34	+33%	< 0.0001

The use of the LLM also led to significant improvements in the intersectionality of the data, illustrating the potential for analysis in higher dimensions that may cause increased cognitive burden for human data collection specialists. To demonstrate this, the perplexity of the dataset, defined as the exponential of Shannon's entropy, was computed across three distinct feature groupings: demographic, voice/clinical, and relevant co‐morbidities [88]. This test represents the effective number of distinct profiles in a hypothetical dataset with uniform distribution, capturing both the count of unique feature combinations and the extent to which these are evenly distributed across patients. As shown in Table 9, the LLM outperformed a random baseline across the three feature groups, including a 101% improvement for demographics (age, sex, ethnicity, BMI), a 232% improvement for voice/clinical (GCS Verbal, FiO_2_, post‐operative status, acute respiratory failure status, model uncertainty status), and a 77% improvement for relevant co‐morbidities (diabetes, immunosuppression).

**TABLE 9:** Comparison of perplexity between LLM‐optimized datasets and 500 counterfactual random sample datasets across three combinations of categorical variables.

Feature combination	LLM dataset	Random dataset	Effect size (%)	*q*
Demographic	88.84 ± 4.01	44.12 ± 3.5	+101%	< 0.001
Clinical/voice	18.95 ± 0.97	5.71 ± 0.46	+232%	< 0.001
Relevant co‐morbidities	3.40 ± 0.06	1.92 ± 0.09	+77%	< 0.001

Table 10 illustrates sample outputs of the pipeline, showcasing the ability of the LLM to select a patient for recruitment and explain the process in terms that are relevant to voice studies. The explainability component may have multiple benefits. For example, data collection specialists could request multiple candidates or ask follow‐up questions to better understand potential problems with the data. Recruitment decisions could then be adjusted by human experts. The second example in Table 10 illustrates a case where the model recommended a candidate with traditionally overrepresented variables but with a clinical profile that would contribute to the robustness of the voice dataset. This may demonstrate the ability of the LLM to evaluate complex, higher‐order interactions between variables, extending beyond what would be captured by conventional evaluations of dataset bias and class imbalances.

**TABLE 10:** Verbatim pipeline outputs showing examples of recruitment decisions made by the LLM based on patient profiles selected from the filtered GOSSIS‐1‐eICU dataset (*n =* 55,589).

*Sample 1*: The candidate (Index 46,564) simultaneously addresses three of the most imbalanced categorical dimensions in the existing voice AI training set: immunosuppression (gap 44.7 pp), ethnicity (Asian, gap 40.7 pp), and post‐operative status (gap 53.7 pp). Adding an immunosuppressed patient introduces physiological changes (e.g., mucosal dryness, altered phonation) that are under‐represented. The Asian ethnicity contributes linguistic and phonetic variation that is scarce in the current cohort. The post‐operative flag adds a scenario of impaired airway protection and potential voice changes from recent surgery. Together, these factors broaden the paralinguistic diversity by providing a voice profile likely to exhibit altered speech coherence, possible hoarseness, and different acoustic patterns, thereby enriching the dataset for more robust voice AI performance across diverse clinical conditions.
*Sample 2*: The patient fills several of the most imbalanced categorical gaps in the current voice AI training set: postoperative = 1 (gap 48.6 pp), FiO_2_ = 1 indicating supplemental oxygen (gap 47.8 pp), and a respiratory Apache body system (under‐represented at 32 patients). Although the ethnicity is Caucasian, the combination of postoperative status, oxygen therapy, and respiratory illness creates a clinical scenario that can markedly alter phonation, breath control, and speech effort, thereby enriching paralinguistic diversity. The profile also includes moderate severity physiologic derangements (elevated heart rate, low‐normal MAP, SpO_2_ 94%) that further diversify voice characteristics associated with acute respiratory compromise.

## 4 Discussion

The AI system described in this report was designed to analyze high‐dimensional patient profiles, ultimately providing more balanced data for training future voice AI models. Results of the study demonstrate the potential for LLM implementation in voice data generation projects that are decentralized, time‐constrained, and high‐volume. The pipeline was able to provide robust recruitment recommendations in scenarios involving multiple possible patients and high‐dimensional clinical data with real‐world missingness patterns. This significantly reduced representation gaps within key dataset attributes. The interaction between the LLM and the uncertainty estimation network may also offer an alternative to binary selection criteria in voice data generation projects. To reduce the high failure rate of clinical AI tools, the composition of training datasets must mirror deployment environments like hospitals or clinics. The system introduced by this work represented health states as model prediction uncertainties and used these values to guide the selection process, resulting in more challenging training datasets compared to the baseline.

There are numerous other benefits that may accompany the implementation of new AI tools for dataset decision support. The ability of LLMs to quickly reason across complex feature sets is one major advantage. Efficiency improvements facilitated by these methods may allow data collection specialists to recruit a larger number of patients or spend more time with each participant. Moreover, when compared to human experts, LLMs may have fewer preconceived biases about which classes are most likely to be unbalanced in the dataset. This is a key consideration given the unique circumstances of each voice data generation project. In the past, these efforts have obtained data from starkly different patient populations around the world, including in India, multiple European countries, the US and Canada, as well as through the mining of social media [15, 16, 17, 18, 89, 90, 91, 92]. With this diversity comes many different potential imbalances that could be overcome by dataset decision support tools.

### 4.1 Limitations

While initial results were promising, this work is a preliminary effort to explore the possibility of dataset decision support tools for voice AI studies. There are multiple limitations that must be considered alongside the contributions. First, the dataset used in this study did not account for demographic categorizations like patients with multiple races. In addition, these results were based on structured EHR data, and the LLM was prompted to make decisions from sets of 50 patients. Real‐world imbalances could be more multimodal, and decisions may need to be made from larger sets of patients. This is particularly true for settings like EDs, where voice data collection is becoming commonplace [39, 40]. More fundamentally, ideal participants may simply be unable or unwilling to participate. Experiments have shown that historically underrepresented patients “perceive healthcare AI to be as untrustworthy and unfair as human medical providers”, potentially reducing willingness to donate data [93]. AI models may also exclude participants due to a lack of necessary context in the EHR and/or implicit biases. LLMs may be an important resource available to data collection specialists, particularly in high‐volume settings, but are not a replacement for human expertise.

Dataset decision support tools should also be evaluated across different types of research. This study focused on the concept of absolute balance, ideal for developing large pre‐training corpora, publicly available datasets, or studies for generalist medical tasks like disease severity evaluation. However, projects for targeted biomarker discovery often have strict inclusion/exclusion criteria, and the requirement for absolute balance may severely reduce the sample size. Small datasets could then limit model generalization capacity.

Finally, the major limitation is that patient profiles from actual voice data generation projects were not used in the simulations. This was due to the lack of available public voice datasets with clear guidelines for LLM research [94]. Generative AI methods should be validated on datasets involving audio recordings.

### 4.2 Future Work

In the future, this concept should be further tested via a researcher‐facing application. To be effective in practice, decision support tools must be designed with an interface that is easy to use in a chaotic environment. Validation studies should include the analysis of metrics such as acoustic variability, phenotypic coverage, signal quality, patient safety, and the performance of audio classifiers trained on resulting datasets. This may uncover new limitations or directions for additional work. To be adopted at scale, LLM decision support tools should demonstrably improve the performance of downstream models without introducing higher levels of noise or increased risks (e.g., by recommending patients unable to safely complete the protocol due to respiratory compromise).

**FIGURE 2:**
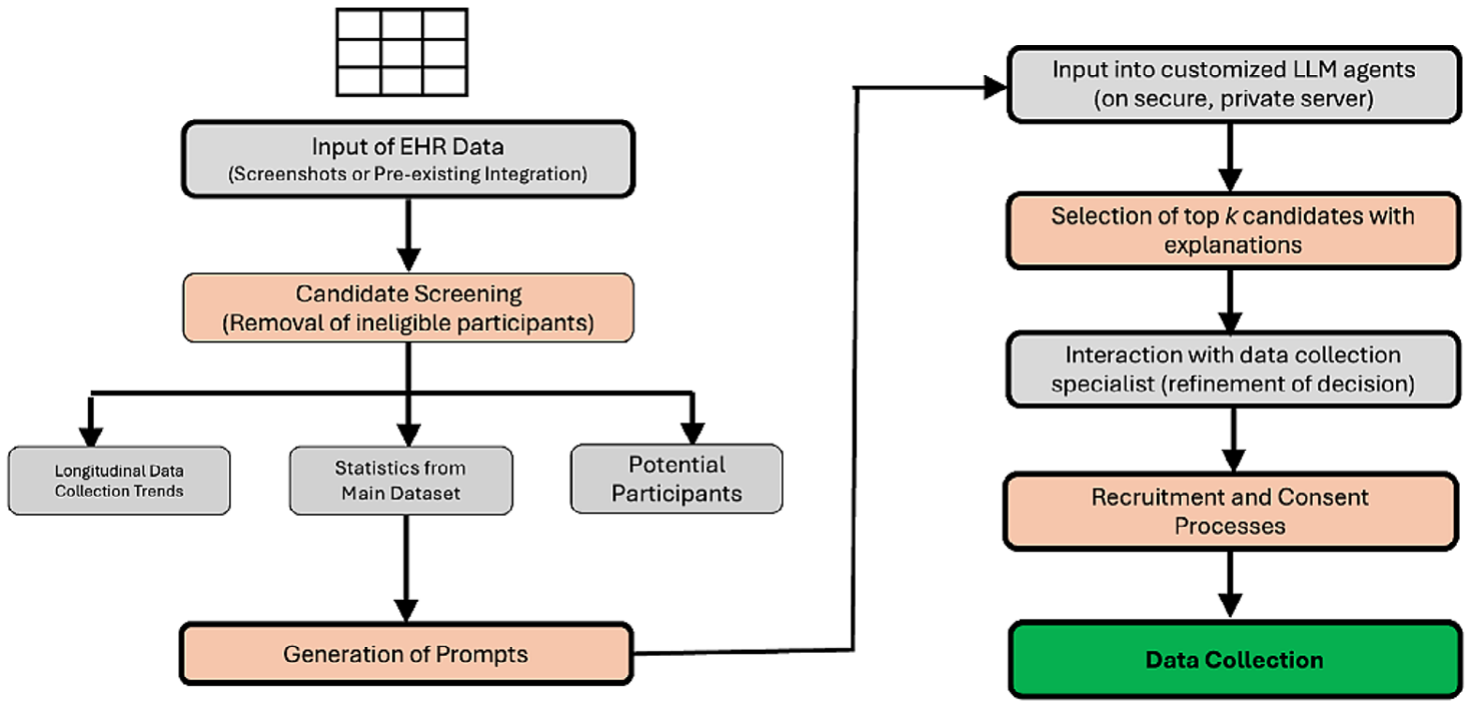
Future implementation of a multi‐agent generative AI system customized for optimizing data collection within voice data generation projects. Steps include (1) input of EHR data via screenshots or existing software integrations, (2) candidate screening to remove patients who may not meet the criteria for the study (e.g., non‐verbal), (3) curation of multiple prompts containing instructions related to class distributions, longitudinal recruitment trends, and possible participants, (4) input into the agent‐based application for assessment, (5) generation of recommendations and explanations, (6) interaction with human experts to refine the decision, (7) recruitment and consent of potential candidates for data collection, and (8) collection of voice data.

Future studies should also involve technical improvements to expand multimodality and scalability. For example, computer vision models may be used to generate recommendations from screenshots or photos of data, reducing the need for cumbersome file uploads in the absence of full integration with EHR software. Multi‐agent systems could also be introduced to increase the number of parallel analyses that could be run by the application. In this expanded framework, one agent may be tasked with examining medication history for potential impacts on the acoustic profile, whereas a different model could assess data collection trends, forecasting future imbalances (Figure 2).

Finally, further work is needed to understand the ethical and privacy implications of prospective voice data collection, which has the potential to contain identifiable information, particularly if unstructured prompts (e.g., “describe your health”) are included in the study protocol. This has been identified as a common concern among stakeholders in voice AI projects [19].

## 5 Conclusion

Transparency and trust are essential for the future adoption of voice AI systems, especially when recommendations may significantly affect patient outcomes. In this report, a simple LLM pipeline was designed for automated recruitment decisions in hospital environments. Expanded versions of this system may offer a new form of decision support that is specifically suited to the needs of data collection specialists. With further validation, LLM tools may enable voice studies to capture a much wider range of biomarkers, improving AI performance.

## Funding

Bridge2AI‐Voice is funded by the NIH Common Fund through the Bridge2AI program, grant number OT2OD032720 and grant number 6128100911.

## Ethics Statement

The study is exempt from institutional review board approval due to the retrospective design, lack of direct patient intervention, and the security schema, for which the re‐identification risk was certified as meeting safe harbor standards by an independent privacy expert (Privacert, Cambridge, MA) (Health Insurance Portability and Accountability Act Certification no. 1031219‐2).

## Conflicts of Interest

The content of this manuscript does not necessarily reflect the views, policies, or opinions of the National Institutes of Health (NIH), the U.S. Government, or the U.S. Department of Health and Human Services. The mention of commercial products, their source, or their use in connection with material reported herein is not to be construed as an actual or implied endorsement by the U.S. government or the NIH.

## Data Availability

The authors have nothing to report.
